# Bullied Adolescent’s Life Satisfaction: Personal Competencies and School Climate as Protective Factors

**DOI:** 10.3389/fpsyg.2019.01691

**Published:** 2019-07-18

**Authors:** Susana Lázaro-Visa, Raquel Palomera, Elena Briones, Andrés A. Fernández-Fuertes, Noelia Fernández-Rouco

**Affiliations:** Department of Education, University of Cantabria, Santander, Spain

**Keywords:** life satisfaction, bullying, personal competencies, school climate, adolescence, self-esteem, emotion regulation, well-being

## Abstract

Although adolescence has been defined as a stage of vulnerability, due to the biopsychosocial changes that happen throughout this developmental stage, it is also one of growth. Some of the core personal competencies that have been identified to promote positive development at this stage while simultaneously preventing risks are: (1) a positive sense of self, (2) self-regulation, (3) decision-making skills, (4) a moral system of belief, and (5) prosocial connectedness. There are many factors and contexts that influence adolescent development. The school climate, for example, has the capacity to promote positive development and life satisfaction, yet on the other hand, it is a context within which different forms of violence, such as bullying, can occur. The principal aim of this study, therefore, is to analyze the influence that bullying has on one’s life satisfaction, while taking into account participants’ socio-demographic characteristics (i.e., gender and developmental stage), their core personal competencies (i.e., problem solving strategies, empathy, emotional repair, self-esteem, and values), and the school climate. To obtain data, a hierarchical regression analysis was conducted with a sample of 647 Spanish students (53.3% female), ranging in age from preadolescence (10–13 years old; 60.3%) to mid-adolescence (14–18 years old; 39.7%), and belonging to diverse socio-economic contexts (15.3% rural) and schools (32.1% public). After gaining informed consent from both the participants and their parents, students completed the survey voluntarily, and under anonymity. Initially results show that gender, developmental stage, and having been bullied were predictors of participants’ levels of life satisfaction. When the core personal competencies were also considered in data analysis process, self-esteem, emotional repair, and social values were those demonstrating significant effects on one’s life satisfaction; moreover, being bullied was a significant predictor too. Finally, after taking school climate into account, only this variable as well as self-esteem and emotional repair were significant predictors of life satisfaction: the other assessed variables were no longer found to be significant predictors (i.e., gender, developmental stage, being bullied, and social values). These results have important implications for education objectives, methodologies, and school functioning: school climate, self-esteem and emotional repair seem to be particularly important for promoting student life satisfaction and for preventing the negative consequences associated with being bullied.

## Introduction

There is a certain consensus in the consideration of subjective well-being as a complex and multidimensional construct created from perceptions, evaluations, and aspirations about one’s own life (Casas, 2011; Kaye-Tzadok et al., 2017). It includes cognitive and affective assessments of people in respect to their own lives, the circumstances affecting them, and the contexts in which they live (Savahl et al., 2018). Subjective well-being can be measured across multiple domains (Campbell et al., 1976; cited in Casas et al., 2014). Among these, life satisfaction expresses the most cognitive component (Kaye-Tzadok et al., 2017), with subjective well-being being the facet on which most research has been focused (Ben-Arieh et al., 2017). Such cognitive and affective evaluations are affected by life circumstances and the social context in which people are living (Savahl et al., 2018).

Despite the increase in publications in recent years about subjective well-being, the amount of research conducted on adolescents is far from the volume of evidence collected on adults (Casas, 2011; Ben-Arieh et al., 2017); also worth noting is that when researching adolescence, more attention has been paid to deficits, risks and problems than to positive development, and growth at this stage (Gademann et al., 2010). Nonetheless, available data consistently show negative relationships between personal well-being and life satisfaction with respect to adolescent problem behaviors, aggressive behavior, or adaptation to school (Lent et al., 2009; Valois et al., 2009). Although the previous studies’ results are inconclusive, a significant decrease in reported levels of life satisfaction between the ages of 11 and 16 (Casas, 2011) can be noted. This could be related to the multiple changes that occur throughout this developmental stage. Females report lower levels of life satisfaction than males, particularly in reference to appearance, bodies, health, free time, and self-confidence (Kaye-Tzadok et al., 2017).

There are various approaches to the relationship among subjective adolescent well-being, personal or social competencies, and the school context. For instance, Guerra and Bradshaw (2008) state that adolescent healthy adjustment is related to five core personal competencies: a positive sense of self, self-control, decision-making skills, a moral system of belief, and prosocial connectedness; in addition, they point out that their promotion “provides a connection between positive youth development and risk prevention programming” (p. 1). Along the same line, previous studies framed in the context of adolescent positive development or in the study of their strengths (see Scales et al., 2016) tend to identify internal and external factors labeled strengths or assets, which play a protective role by increasing the probability of developing healthy trajectories in adolescence (Curran and Wexler, 2017). Internal factors include future expectations, self-control and decision-making (Curran and Wexler, 2017), social competence, positive values, self-esteem, and self-regulation (Calmeiro et al., 2018). The relationship between empathy and well-being has scarcely been demonstrated in studies with youth (Shanafelt et al., 2005) and adolescent subjects. In their work regarding assets and subjective quality of life, Valois et al. (2009) indicate that adolescents with lower levels of empathy present lower levels of life satisfaction. Recently, Taylor et al. (2017) have organized these competencies more systematically into self-awareness (e.g., recognition of emotions and values), self-management (e.g., emotional regulation), social awareness (e.g., empathy), relationship skills, and responsible decision making. These competencies appear to be related to success in both school and life in general. Some of these variables have come to light in studies on resilience as well, highlighting factors that could facilitate adolescent adaptation despite their having lived through extreme risk situations, such as violence (Hinduja and Patchin, 2017). Few studies, however, explore the relationship between these competencies and life satisfaction (Valois et al., 2009). Among the contextual variables, we know that school climate is considered to be one of the most significant predictors of student psychological adjustment in general (Voight and Nation, 2016), specifically related to the social climate. Schools that promote the positive development of their pupils are characterized by their ability to facilitate positive links between pupils and teachers. This is done by promoting a climate of affection and security with clear rules and limits via affective proximity in interpersonal relations, and finally by fostering student participation and a feeling of belonging to the center, thereby generating opportunities for the development of personal, and social-emotional skills (Oliva et al., 2011b; Pertegal and Hernando, 2015). It is associated with adolescent well-being, academic outcomes, lower levels of bullying behavior, and positive attitudes toward interpersonal violence (Bradshaw et al., 2013; Espelage et al., 2014; Low and Van Ryzin, 2014; Benbenishty et al., 2016; Konishi et al., 2017; Rezapour et al., 2019). In turn, the results show that improvements made to the school climate correspond to greater awareness of the issue of bullying (Waasdorp et al., 2012), whereas a perceived hostile school climate corresponds to less intervention in bullying situations (Yoon et al., 2016).

We are aware, on the other hand, that subjective well-being can be affected by various situations experienced by adolescents, including school victimization (Savahl et al., 2018; Zych et al., 2018; Varela et al., 2019). Bullying is defined as “a phenomenon of intentional aggression by one or more persons upon another or others in a way that is both repeated and maintained over time, and in which a power imbalance exists between the aggressor and the victim” (Olweus, 1993, p. 9). There are developmental variations in bullying behaviors: its prevalence seems to increase in late elementary school, peak in middle school, and decline in high school, although certain forms, such us cyberbullying may increase in high school (Olweus, 1993; Bradshaw et al., 2007). Research carried out in recent decades shows that bullying significantly affects the health of victims. Menesini and Salmivalli (2017) have reviewed a number of studies carried out in different countries: the results show negative physical, psychological, relational and general well-being consequences, and highlight the relationship between these and poor school achievement, loneliness, and the internalization of problems. The deterioration of mental health in victims of bullying is especially reflected by an increased risk of anxiety and depression (Ttofi et al., 2011; Evans and Smokowski, 2015), as well as behavioral difficulties such as increased criminal behavior (Sapouna and Wolke, 2013).

The study of bullying in relation to perceived life satisfaction has only recently begun to be addressed. Available results show that bullying tends to have a negative impact on subjective adolescent well-being: lower levels of life satisfaction tends to be observed in adolescents who have suffered cyberbullying as opposed to those who have not been bullied (Arnarsson et al., 2019), as well as lower average levels of well-being in preadolescent bullying victims, with victimization being the variable posing the greatest negative impact to well-being (Alcántara et al., 2019). Similarly, Savahl et al. (2018) show the negative relationship between subjective well-being and bullying in preadolescents from different countries.

Recently, some studies have incorporated the analysis of the protective role that contextual and personal variables play on the well-being of bullied adolescents, as stated in the review study carried out by Zych et al. (2019). Among the personal variables studied, Casas et al. (2015) point out to the role of emotional intelligence as a possible protective factor against face to face bullying, finding that high levels of emotional attention and low levels of clarity and reparation are able to anticipate victimization. Likewise, Elipe et al. (2012) found that victims exhibited higher levels of emotional attention and lower levels of emotional reparation. These results do not appear to be conclusive given that subsequent studies found no significant differences with respect to clarity and emotional reparation, though greater emotional attention was in fact observed among victims of bullying (Beltrán-Catalán et al., 2018). Personal factors such as prosocial connectedness, self-esteem, self-concept, and decision-making skills are pointed out as protector variables against victimization in the review study of 18 meta-analyzes (Zych et al., 2019). Jackson et al. (2017) point out the complex relationship between protective factors and victimization, emphasizing the interaction that exists between individual, and contextual factors. Factors such as gender and personality type could mediate the relationship between individual protective factors and being bullied. Overbeek et al. (2010) highlight the protective role that self-esteem plays in victims of bullying, though this is mediated by the personality type of the adolescent – presenting as a predictor for over-controlling adolescents. The results of Sapouna and Wolke (2013) indicate that a high self-esteem and a high social connection are related to emotional and behavioral resilience in adolescent victims of bullying; in addition, males demonstrate greater emotional resilience while females demonstrate greater behavioral resilience. There are even fewer results regarding the protective role that empathy could potentially play among victims of bullying. The results regarding the association between empathy and the role of the aggressor have been contradictory (Jolliffe and Farrington, 2006), although most studies conclude that there is no relationship between representing high levels of empathy and being bullied (Zych et al., 2019). Moreover, while there are more studies focused on risk factors that lead to bullying, studies attempting to highlight the core personal competencies as protective factors against bullying in adolescence are rather limited in current literature (Méndez et al., 2017; Zych et al., 2018).

Among the contextual variables, the protective role of the school climate as it relates to the well-being of adolescent victims of bullying has been addressed. Miranda et al. (2019) show how the support received from adults within the context of the home and school allows for improved levels of well-being in adolescent victims of bullying, as opposed to those who perceive less support. This is especially true when the support, or lack thereof, stems from the family nucleus. Other results show that regardless of the school climate, being a victim of bullying in adolescence is associated with lower levels of well-being as compared to those who have not experienced this situation, although general well-being is lower in adolescents who perceive the school climate to be worse (Alcántara et al., 2019). The complex relationship between school climate and bullying does, nevertheless, need to be clarified (Bradshaw et al., 2013; Acosta et al., 2019).

In conclusion, although the personal competencies and school climate can play an important role in fostering well-being, at present there are still very few results on the extent of the protective role they play in the subjective well-being of adolescent bullying victims. In this paper we explore the approach to core personal competencies based on Guerra and Bradshaw’s (2008) proposal, which defines five basic competencies essential for healthy adjustment in adolescents: a positive sense of self, self-regulation, decision-making skills, a moral system of belief, and prosocial connectedness. The central assumption is that those who are more skilled in self-management and interpersonal relationships would be less willing to be involved in risky behaviors throughout childhood and adolescence (Tolan et al., 2016). Although school climate dimensions may be organized differently in the various theoretical approaches (Konishi et al., 2017), in this study it will be considered from a more social perspective, including only the most common dimensions that have been found to be closely related to academics, sociability, physical security and affective bonding, and well-being in early adolescence (Lester and Cross, 2015).

Currently, it is not yet clear what role school climate may have on the subjective well-being of adolescent bullying victims, particularly when taking into account the personal competencies of adolescents. Research underlines, on the one hand, that academic proficiency is important for positive adolescent development, yet on the other hand, it highlights that such proficiency is negatively associated with greater internalized, and externalized problems throughout this stage (Pertegal et al., 2010). Despite this, few studies look specifically at these findings in relation to subjective well-being and school violence, or more specifically, in relation to bullying. Existing research does not consider, furthermore, what happens during preadolescence. That is to say that early adolescence and preadolescence are not incorporated into the research, which is especially remarkable considering that research on bullying and cyberbullying is extensive both nationally and internationally.

Taking the background into account, this research aims to analyze the impact that being bullied has on the life satisfaction of a sample of students between 10 and 18 years old. In addition, given that they would undoubtedly influence life satisfaction, certain variables will be also taken into account: the socio-demographic characteristics (i.e., gender and developmental stage) of select participants, certain personal factors (i.e., the core personal competencies: problem solving strategies, empathy, emotional repair, self-esteem, and values), as well as one contextual factor (i.e., the school climate).

## Materials and Methods

### Participants

A total of seven Spanish schools selected by incidental sampling participated in the study with the following socio-economic environments taken into consideration: rural (15.3% of the sample), semi-urban (42.1%), and urban (42.6%). The sample is composed of a total of 693 students (mean age: 12.98, SD: 1.78) from the last cycle of primary education (10–13 years; 60.3% preadolescents) and secondary education (14–18 years; 39.7% adolescents), balanced by gender (women: 53.3%; men: 43.8%).

### Instruments

#### Socio-Demographic Questionnaire

Gender, course, and date of birth were first asked to fill.

#### European Bullying Intervention Project Questionnaire (EBIPQ; Ortega-Ruiz et al., 2016)

This is an instrument composed by 14 items to measure bullying: half evaluate aggressions suffered as a victim (bullied) and the other half those committed as an aggressor (buller). The items refer to aggressions such as hitting, insulting, threatening, stealing, profanity, exclusion, or the spreading of rumors. The elements present a response modality that graduates the frequency of occurrence of the behaviors that took place in the last 2 months (0 = Never; 1 = Yes, once or twice; 2 = Yes, once or twice a month; 3 = Yes, about once a week; and 4 = Yes, more than once a week). In this work we have only used the victim variable. The reliability of this instrument in the validation study was adequate as it was in this study (alpha = 0.80).

#### Social Attitudes and Cognitive Strategies (SACS; Moraleda et al., 2004)

This instrument evaluates 9 social attitudes and 10 social thinking strategies among 12–17 year old students. Within the strategies of social thinking, we have used a comprehensive score to measure decision making skills, more specifically, problem solving strategies, which is derived from four subscales corresponding to the four steps of the process: observation and retention of relevant information regarding social situations, search for alternative solutions to resolve social conflict, anticipation, and understanding of the consequences that may result from social behavior, and selection of appropriate means for the ends pursued in social conduct. The adolescents indicated via a 7-point likert scale the extent to which they agree with the content of the sentence. The original study showed adequate reliability across the board as it did here (alpha = 0.82).

#### Basic Empathy Scale (BES; Jolliffe and Farrington, 2006)

In order to assess prosocial connectedness, the scale selected for this study is the 9-item shortened version of the original 20-item scale, that was validated for use in the Spanish adolescent population by Oliva et al. (2011a). The items are distributed in two scales, one corresponding to affective empathy, comprised of four items; and another corresponding to cognitive empathy with the remaining items. Adolescents responded on a 5-point likert scale based on the extent to which they agree with the proposed statements. The internal consistency of the validation complies with satisfactory psychometric properties, here the reliability to affective empathy (alpha = 0.77) and cognitive empathy (alpha = 0.73), and to full scale (alpha = 0.79) seem adequate.

#### Trait Meta Mood Scale (TMMS; Salovey et al., 1995)

This twenty-four-item questionnaire is designed to assess the behavioral trends and perceptions that people have regarding their ability to deal with emotions, the clarity with which they perceive those emotions, and their ability to repair their emotional states. In this study we have used only the reparation of negative emotional states subscale (e.g., “Although I feel bad, I will do my best to think happy thoughts”) to assess self-regulation competence. Subjects had to assess each assertion about themselves with a likert scale of 1–5 that represents their level of agreement with each item. The scale has been adapted and validated for a Spanish adolescent population (Salguero et al., 2010), and it has demonstrated adequate internal consistency. The alpha reliability of emotional repair subscale in this study was 0.84.

#### Rosenberg Self Esteem Scale (RSS; Rosenberg, 1965)

The Spanish teen adaptation of Atienza et al. (2000) has been used, exhibiting adequate psychometric properties. It consists of 10 items which evaluate the overall assessment of positive sense of self, specifically self-esteem as it relates to feelings of respect, and acceptance of oneself (e.g., “I am capable of doing things as well as other people”) which are scored using a 4-point likert scale according to the extent of accordance. Its reliability in this study, as well as in the original version, was adequate (alpha = 0.83).

#### Values for Adolescent Positive Development Scale (VAPDS; Antolín et al., 2011)

This scale assesses participants’ moral system of belief through asking the importance that adolescents attach to a set of values associated with positive development at this stage. It consists of 24 items grouped into 8 subscales: social commitment, prosociality, justice and equality, honesty, integrity, responsibility, social recognition, and hedonism. These are further grouped into three scales: social values, personal values, and individualistic values. The students had to answer by means of a 7-point likert scale indicating the degree of importance they attach to each sentence (1 = not at all important, 7 = extremely important). In the original study, adequate reliability and validity of the scales was achieved, as well as in this study (alpha were 0.88, 0.84, and 0.77, respectively).

#### School Climate and Functioning Scale (SCFS; Oliva et al., 2011a)

The version which measures student perception has been used. This likert-7 scale (i.e., 1 = totally false, 7 = totally true), is made up of 30 items that measure four factors related to the climate and functioning of the school: climate, which includes the degree to which pupils perceive the relationships among peers as good, and the perception of the school climate as safe (e.g., “there is a good rapport among everyone in this school”); links, which, on the one hand, assess the degree to which students have a feeling of belonging and satisfaction with their school and, on the other hand, assess perceived teacher support (e.g., "teachers are available to address each student’s individual doubts"); clarity of norms and values that, on the one hand, analyses the degree to which students perceive the limits to be clear with regard to existing school norms, and, on the other hand, quantifies the perception of coherence in the promotion of school values on the part of teachers (e.g., “all students know the limits and rules that must be respected in this centre”); and empowerment and positive opportunities, which involves valuing the school’s resources and facilities as opportunities, offering activities aimed at the students along with their perception of the influence these activities have on the life of the school, or of participation being fostered (e.g., “when there is a conflict, students have an opinion and participate in its resolution”). The instrument allows the use of either a global score (i.e., the average of scores across the four factors), or multiple scores applied from the individual factor: in this work the former was chosen. In its original version, the said global factor obtained high reliability as it is in our study (alpha = 0.79).

#### Personal Well-Being Index (Cummins et al., 2003)

This scale is made up of seven items of which a person uses an eleven-point scale (where 0 = completely dissatisfied and 10 = completely satisfied) to evaluate, in a relatively generic and abstract way, degree of satisfaction with a given aspect of life. A global life satisfaction factor is obtained, in this case by averaging the scores. It has been tested with a Spanish population beginning at 12 years of age, and demonstrates good psychometric properties (see Casas et al., 2011) as it was in this study (alpha = 0.86).

### Procedure

The study was carried out in accordance with the Declaration of Helsinki. Ethics approval was obtained from the University of Cantabria Research Projects Ethics Committee. Data was collected upon obtaining informed and written consent from all participating student’s parents and the students themselves. Throughout the course of a 50-min classroom session and in the presence of a researcher, the students were reminded of the study objectives and were asked to voluntarily and anonymously fill in the various questionnaires, acknowledging that they would be free to withdraw from the study at any time. All the instruments used have proved to be valid and reliable within the Spanish population, both in their original version and in their adaptations, when applied.

### Data Analysis

A correlational study was carried out with the aim of finding results according to the proposed objectives. We first proceeded to dichotomize the variable of the victim of bullying to distinguish the students who had not suffered bullying (71.6%) from those who had (28.4%). This variable has been categorized using the third quartile, which implies that it has been scored at least in two items and in the case of one of them, with high frequency (4 or 5), or in five items with minimal frequency (2), in line with expert consensus on frequency of occurrence (Nansel et al., 2001; Solberg and Olweus, 2003; Volk et al., 2006; quoted in Díaz-Aguado et al., 2013). We then proceeded to standardize those variables measured with Likert scales using the *z*-score formula. Comparing scores is easier in this way, given that different scales were used in their measurement. Cohen et al. (2003), furthermore, have recommended this procedure to carry out hierarchical regression analysis in order to maintain unambiguous interpretations of the effects. After the descriptive analyses, we carried out correlation analyses depending on the type of variable used, identifying correlations among the variables being studied that proved significant (Table 1). Of the correlation analyses selected were: Pearson (relationship between linearly related variables), Biserial-punctual (conducted with the Pearson correlation formula with the exception that one of the variables is dichotomous) or Phi (relationship between two dichotomous variables). A hierarchical step-by-step regression analysis was then carried out (Gelman and Hill, 2006) to determine the effects the different variables studied had on life satisfaction (criterion variable). In the first step, developmental stage, gender, and being bullied were introduced. In the second step, the core personal competencies were inserted: problem solving strategies, empathy (affective and cognitive), emotional repair, self-esteem, and values (social, personal and individualistic). Finally, school climate was entered in the third step.

**TABLE 1:** Correlations and descriptive statistics of study variables.

**Variable**	**1**	**2**	**3**	**4**	**5**	**6**	**7**	**8**	**9**	**10**	**11**	**12**	**13**
1. Developmental stage													
2. Gender	0.09^*^												
3. Bullied	–0.05	0.02											
4. Problem solving	−0.08^*^	0.10^*^	–0.17^∗∗∗^										
5. Affective empathy	–0.03	0.28^∗∗∗^	0.05	−0.08^*^									
6. Cognitve empathy	0.03	0.15^∗∗∗^	0.08^*^	–0.04	0.41^∗∗∗^								
7. Emotional repair	–0.19^∗∗∗^	−0.10^*^	−0.07^*^	0.09^*^	0.17^∗∗∗^	0.28^∗∗∗^							
8. Self-esteem	–0.16^∗∗∗^	–0.06	–0.27^∗∗∗^	0.25^∗∗∗^	–0.01	0.10^*^	0.44^∗∗∗^						
9. Social values	–0.17^∗∗∗^	0.14^∗∗∗^	–0.05	0.04	0.36^∗∗∗^	0.33^∗∗∗^	0.34^∗∗∗^	0.10^∗∗^					
10. Personal values	–0.05	0.13^∗∗^	−0.09^*^	–0.01	0.23^∗∗∗^	0.28^∗∗∗^	0.28^∗∗∗^	0.20^∗∗∗^	0.67^∗∗∗^				
11. Individualistic values	–0.02	–0.01	0.09^*^	–0.30^∗∗∗^	0.10^∗∗^	0.07	0.15^∗∗∗^	0.11^∗∗^	0.29^∗∗∗^	0.37^∗∗∗^			
12. School climate	–0.26^∗∗∗^	–0.06	–0.23^∗∗∗^	0.12^∗∗^	0.09^*^	0.13^∗∗^	0.34^∗∗∗^	0.37^∗∗∗^	0.32^∗∗∗^	0.34^∗∗∗^	0.11^∗∗^		
13. Life satisfaction	–0.15^∗∗∗^	−0.08^*^	–0.22^∗∗∗^	0.12^∗∗^	0.07	0.14^∗∗∗^	0.42^∗∗∗^	0.56^∗∗∗^	0.22^∗∗∗^	0.22^∗∗∗^	0.15^∗∗∗^	0.46^∗∗∗^	
Mean	0.40	0.55	0.28	0.00	0.00	0.01	–0.00	–0.01	0.01	0.01	0.01	0.01	–0.00
SD	0.49	0.50	0.45	0.99	1.00	1.00	0.99	0.98	1.00	0.99	1.00	1.00	0.98

^∗∗∗^*p* < 0.001; ^∗∗^*p* < 0.01; ^*^p < 0.05. Developmental stage (0, preadolescence; 1, adolescence), gender (0, male; 1, female) and bullied (0, not bullied; 1, bullied) are dichotomized variables, the scores of the other variables were rescaled using the *z*-score formula. These standardized variables have a mean of zero and a SD of 1.

## Results

### Preliminary Analysis

Table 1 presents the correlations among the variables of the study. As expected, life satisfaction correlates positively and significantly with the core personal competences with the exception of affective empathy, with which no significant relationship was found. As for the correlations between the variables that make up the core personal competences (i.e., problem solving strategies, cognitive, and affective empathy, emotional repair, self-esteem, social, personal, and individualistic values), all were found to relate positively and significantly to each other, with the exception of problem solving strategies which was found to be negatively related to affective empathy and individualistic values, and had no relation to cognitive empathy, social, or personal values. Affective empathy showed no association with self-esteem, nor was a relation established between cognitive empathy, and individualistic values.

On the other hand, the stage presents a significant and negative relationship with that of problem solving strategies, emotional repair, self-esteem, social values, climate, and personal well-being: older participants present lower scores in these variables. Gender, on the other hand, is significantly and positively related to problem solving strategies, affective and cognitive empathy, and social and personal values. This means that females score higher in these variables. Gender showed a significant and negative relationship as well with emotional repair and life satisfaction; with males scoring higher in both variables in this case. As for being a victim of bullying, the significant and negative correlation with problem solving strategies, emotional repair, self-esteem, personal values, school climate and personal well-being can be seen, with those who have suffered bullying being the ones who score the lowest among these variables. In addition, being a victim of bullying correlates significantly and positively with cognitive empathy and with individualistic values; that is, bullying victims score higher on cognitive empathy, and individualist values.

### Regression Analysis

A hierarchical regression analysis has been performed to predict life satisfaction. The main effects of this stage, gender and bullying, were the predictor variables in the first step. In the second step, variables related to personal competencies (i.e., problem solving strategies, cognitive and affective empathy, emotional repair, self-esteem and personal, social, and individualistic values) were added to the regression. In step 3, the school climate variable was introduced. The results are presented in Table 2.

**TABLE 2:** Hierarchical multiple regression analysis: Standardized regression coefficients.

**Predictor variable**	**Life satisfaction**		
	**Step 1**	**Step 2**	**Step 3**
Developmental stage (0, preadolescence; 1, adolescence)	–0.15^∗∗∗^	–0.04	–0.01
Gender (0, male; 1, female)	−0.07^*^	–0.06	–0.05
Victim (0, not bullied; 1, bullied)	–0.22^∗∗∗^	–0.10^∗∗^	–0.06
Problem solving		–0.01	–0.02
Affective empathy		0.02	0.01
Cognitive empathy		0.02	0.02
Emotional repair		0.14^∗∗∗^	0.11^∗∗^
Self-esteem		0.43^∗∗∗^	0.38^∗∗∗^
Social values		0.11^*^	0.08
Personal values		0.01	–0.04
Individualistic values		0.05	0.05
School climate			0.26^∗∗∗^
*R*^2^	0.03	0.36	0.41
*R*^2^ change	0.08	0.29	0.05

^∗∗∗^*p* < 0.001; ^∗∗^*p* < 0.01; ^*^p < 0.05.

Step 1 shows that greater life satisfaction is predicted by not having been bullied, being preadolescent, and being male. The variables in step 2 explain an additional 28% of the variance in life satisfaction: both emotional repair and self-esteem as well as social values have the expected positive effect on life satisfaction. Finally, the results of step 3 indicate that, when the school climate is taken into account in the life satisfaction prediction, only the positive effects of emotional repair and self-esteem remain consistent, while the effects of the stage in question, gender, not having been bullied, and even social values appear to have vanished. The complete model explains 41% of the variance in life satisfaction.

## Discussion

This paper was focused on predicting life satisfaction of students (male and female) enrolled in compulsory education (the last cycle of primary education, preadolescents; and secondary education, adolescents), who could have suffered from bullying. With this purpose, the predictive power of some personal factors (i.e., five core personal competencies: problem solving strategies, empathy, emotional repair, self-esteem, and values) and some contextual ones (i.e., the school climate), was analyzed. All these factors are known to be essential to healthy adjustment (Guerra and Bradshaw, 2008; Voight and Nation, 2016). Nonetheless, as previously stated, no strong line of research exists in regards to the multidimensional approach proposed by Guerra and Bradshaw (2008). For instance, there little research has been conducted on the core personal competencies invariance across age groups, the interrelations among these competencies, or their predictive power in specific situations such as having been bullied (Tolan et al., 2016; Zych et al., 2018).

The main results of this study demonstrated that a negative impact on life satisfaction (i.e., a key component of subjective well-being) is more likely if you: have been a victim of bullying (vs. not having been a victim), are an adolescent (vs. preadolescent), and are female (vs. male). When the core personal competencies were taken into consideration to account for changes in life satisfaction, being bullied remained as a significant predictor, while self-esteem, emotional repair and social values emerged newly as significant predictors, too. Finally, after introducing school climate in the regression analysis, self-esteem and emotional repair continued being predictors of life satisfaction, and school climate emerged as a predictor too.

Previous literature pointed out that individuals with high core personal competencies might present higher levels of life satisfaction due to the frequency of having experienced pleasant or positive emotions, that is to say, people with better core competencies are more satisfied because they experience positive emotions more frequently that those who do not (Extremera and Rey, 2018). In addition, the core personal competencies are foundational to a wide array of positive developmental processes and are essential to healthy development and thriving (Metzger et al., 2018). School climate is one of the most relevant aspects for psychological health in students (Voight and Nation, 2016), associated not only with well-being, but also with bullying behavior and attitudes against it (Benbenishty et al., 2016; Konishi et al., 2017).

These results are consistent with those recently presented regarding the negative impact that being bullied has on one’s well-being (Arnarsson et al., 2019). Previous studies have identified a decrease in life satisfaction throughout adolescence (Harris et al., 2018), with an even greater impact among those who have been a victim of violence (Olweus and Breivik, 2014), and especially those who are female. With respect to this last conclusion, it has been determined that among females, life satisfaction depends more on having a quality social support (Saphire-Bernstein and Taylor, 2013). It has been found, furthermore, that girls who were bullied tend to show lower levels of well-being than boys (Siegel et al., 2009). Therefore, the results of this work seem to point out a situation of intersectionality in the explanation of life satisfaction.

In reference to subjective well-being, generally speaking, the positive relationship between this variable and the personal core competencies is supported by previous studies (Valois et al., 2009; Curran and Wexler, 2017). In present research the relationship is more vague in the case of empathy, which does not appear to be related to life satisfaction (i.e., affective empathy) or in which the correlation is weak (i.e., cognitive empathy) (Table 1). In the few studies that have taken this variable into account when assessing adolescent or youth life satisfaction, those participants who were perceived as less empathetic also demonstrated lower levels of life satisfaction (Valois et al., 2009; Lachmann et al., 2018). Nevertheless, it has been suggested that empathy could predict adolescents’ life satisfaction only indirectly through positive and negative emotions (Lu et al., 2019), variables that were not assessed in this study. When core competencies are not activated, however, and when one resorts, for example, to the use of preventative (and not proactive) coping strategies during hardship, satisfaction with life tends to be lower (Lyons et al., 2016).

Regarding the core personal competencies and consistent with previous works (Sánchez-Álvarez et al., 2015; Calmeiro et al., 2018), self-esteem, emotional repair, and social values demonstrated the expected positive effect on life satisfaction; however, as previously stated, social values cease to be significant when the school climate is introduced in the regression. It is interesting to note that some core personal competencies did not prove to be predictors of life satisfaction in the sample studied (i.e., problem solving strategies, cognitive empathy, affective empathy, personal values, and individualist values). Given that previous studies have determined these relationships (Bobowik et al., 2011; Cenkseven-Önder and Çolakkadioglu, 2013; Schipper and Petermann, 2013; Choi et al., 2016; Lachmann et al., 2018), it could be deduced that emotional repair, self-esteem and social values prevail over the explanatory contribution of other variables. In particular, self-esteem presents the greatest positive relationship with subjective well-being; in fact, the relationship between adolescent self-esteem and satisfaction with life has been the object of recurrent study, with a solid positive relationship having been established between the two (Campbell, 1981; Palacios et al., 2015). Emotional repair and social values are also important predictors of life satisfaction among the sample studied; perhaps this is due to the fact that adolescent life satisfaction is more closely related to the use of adaptive strategies such as the ability to regulate one’s emotional state, and social values (e.g., other-oriented goals), as has been previously suggested (Sánchez-Álvarez et al., 2015; Blau et al., 2018; Rey et al., 2019). However, this assertion should be verified via additional studies.

Finally, in previous studies school climate and life satisfaction were found to be negatively related to victimization (Martínez-Ferrer et al., 2011), and our results seem to confirm that the perceived school climate is an aspect of particular relevance of students life satisfaction (Suldo et al., 2013), including in situations in which someone is being bullied; the help and support of peers and teachers could prevent incidences of bullying, and as well as diminish the negative effect that bullying has on well-being (Flaspohler et al., 2009; Miranda et al., 2019; Rezapour et al., 2019; Varela et al., 2019; Zych et al., 2019).

On a practical level, these findings suggest the importance of considering the overall context and not just individual aspects, interpreting it as a network of elements that work in synergy, though at the same time poses some challenges. In this respect, the results of this work illustrate the importance of taking care of minors’ self-esteem and their capacity for emotional recovery without disregarding the role that school climate plays. Lastly, the importance of teachers, parents and peers in preventing and/or stopping bullying experiences, as well as in mitigating the negative impact of being bullied on life satisfaction (Sung et al., 2018; Miranda et al., 2019; Zych et al., 2019) is recognized; as Savahl et al. (2018) pointed out, “practitioners, teachers, and caregivers of children need to be aware that even though children may demonstrate reasonable levels of life satisfaction, they may be victims of bullying, and thus at risk for the associated negative outcomes” (p. 15). Finally, having effective social support might directly result in increased levels of well-being. Development of emotional skills increases not only current, but also future well-being, given that having more personal resources tends to result in an increase of social support, as was concluded in previous research dealing with older populations (Rey et al., 2019).

### Limitations and Future Directions

In interpreting the results of this work, certain limitations need to be taken into account. The use of self-reports as a method of data collection may increase response bias, though this is the most common form of information collection in this area, and among this population (Gilman and Huebner, 2003). While the use of multiple sources of information (e.g., teachers, parents, and peers, etc.) would address this constraint, though this analytical strategy was not possible given the design of the current study. Moreover, in this research the core personal competencies were measured separately by different instruments and without calculating the total score in the construct, as has been done previously (e.g., Denham et al., 2009): design of an instrument to assess the core personal competencies as a construct would accordingly be beneficial in future studies. In addition, transversal design prevents us from being able to make explanatory causal statements over time; it does, however, allow us to establish relationships at a given point in time between variables that have not yet been explored. Likewise, the type of sampling carried out dictates that potential generalizations of these results must be made with caution: it is unclear if these findings are applicable to other samples of students or autonomous communities of Spain. Finally, not having qualitative data makes in-depth analysis of some of the results obtained quite difficult, especially with respect to their origin. All these issues should be addressed through additional studies.

Despite its limitations, this work contributes to the scientific literature in that (1) it establishes a connection between the core personal competencies, the school climate, and life satisfaction, both for those who are victims of bullying and those who are not, as well as for both males and females in their final cycle of primary school (preadolescents) and secondary school (adolescents); (2) it emphasizes the importance of emotional repair and self-esteem in the explanation of life satisfaction, which thereby offers vital information for the development of educational actions; and (3) it highlights the role of the school climate, evidencing the important function of the school as a community for the promotion of life satisfaction from within the school climate.

These results point to future trends insofar as they emphasize the need to promote a positive school climate, which inevitably involves the entire educational community, while meanwhile calling on institutions to make policy decisions that point in this direction. This would contribute not only to healthier environments, but also to more satisfied citizens. In keeping with the above, it would be beneficial to develop work that incorporates other voices, such as those of teachers, administrative staff, management teams, and families in order to have a more comprehensive view of the educational community. At the same time, longitudinal studies would make it possible to explore the way such a community functions over time.

## Data Availability

The raw data supporting the conclusions of this manuscript will be made available by the authors, without undue reservation, to any qualified researcher.

## Ethics Statement

This study was carried out in accordance with the Declaration of Helsinki. Ethics approval was obtained from the University Research Ethics Committee. Data was collected upon obtaining informed written consent from participating centers, families, and the students themselves. Throughout the course of a 50-min classroom session and in the presence of a researcher, the students were reminded of the study objectives and were asked to voluntarily and anonymously fill in the various questionnaires, acknowledging that they would be free to withdraw from the study at any time.

## Author Contributions

All authors were involved in data acquisition. SL-V and RP carried out the work. SL-V wrote and revised the first draft of the manuscript. RP and EB analyzed and described the data. AF-F and NF-R explained the results. All authors revised the work critically for important intellectual content, and approved the final version to be published.

## Conflict of Interest Statement

The authors declare that the research was conducted in the absence of any commercial or financial relationships that could be construed as a potential conflict of interest.

## References

[B1] Acosta J. Chinman M. Ebener P. Malone P. Phillips A. Wilks A. (2019). Understanding the relationship between perceived school climate and bullying: a mediator analysis. *J. Sch. Violence* 18 200–215. 10.1080/15388220.2018.1453820 30778279 PMC6377176

[B2] Alcántara S. C. González-Carrasco M. Montserrat C. Casas F. Viñas-Poch F. Pereira D. (2019). Peer violence, school environment and developmental contexts: its effects on well-being. *Ciên. Saúde Colet.* 24 509–522. 10.1590/1413-81232018242.013020171 30726383

[B3] Antolín L. Oliva A. Pertegal M. A. López-Jiménez A. M. (2011). Desarrollo y validación de una escala de valores para el desarrollo positivo adolescente. *Psicothema* 23 153–159. 10.1037/t16329-000 21266157

[B4] Arnarsson A. Nygren J. Nyholm M. Torsheim T. Augustine L. Bjereld Y. (2019). Cyberbullying and traditional bullying among Nordic adolescents and their impact on life satisfaction. *Scand. J. Public Health* 10.1177/1403494818817411 [Epub ahead of print]. 30672390

[B5] Atienza F. L. Moreno Y. Balaguer I. (2000). Análisis de la dimensionalidad de la Escala de Autoestima de Rosenberg en una muestra de adolescentes valencianos. *Rev. Psicol. Univ. Tarraconensis* 22 29–42.

[B6] Beltrán-Catalán M. Zych I. Ortega-Ruiz R. Llorent V. J. (2018). Victimisation through bullying and cyberbullying: emotional intelligence, severity of victimisation and technology use in different types of victims. *Psicothema* 30 183–188. 10.7334/psicothema2017.313 29694319

[B7] Ben-Arieh A. Dinisman T. Rees G. (2017). A comparative view of children’s subjective well-being: findings from the second wave of the ISCWeB project. *Child. Youth Serv. Rev.* 80 1–2. 10.1016/j.childyouth.2017.06.068

[B8] Benbenishty R. Avi-Astor R. Roziner I. Wrabel S. L. (2016). Testing the causal links between school climate, school violence, and school academic performance: a cross-lagged panel autoregressive model. *Educ. Res.* 45 197–206. 10.3102/0013189X16644603

[B9] Blau I. Goldberg S. Benolol N. (2018). Purpose and life satisfaction during adolescence: the role of meaning in life, social support, and problematic digital use. *J. Youth Stud.* 10.1080/13676261.2018.1551614

[B10] Bobowik M. Basabe N. Páez D. Jiménez A. Bilbao M. A. (2011). Personal values and well-being among Europeans, Spanish natives and immigrants to Spain: does the culture matter? *J. Happiness Stud.* 12 401–419. 10.1007/s10902-010-9202-1

[B11] Bradshaw C. P. Sawyer A. L. O’Brennan L. M. (2007). Bullying and peer victimization at school: perceptual differences between students and school staff. *Sch. Psychol. Rev.* 36 361–382. 21552337

[B12] Bradshaw C. P. Waasdorp T. E. Goldweber A. Johnson S. L. (2013). Bullies, gangs, drugs, and school: understanding the overlap and the role of ethnicity and urbanicity. *J. Youth Adolesc.* 42 220–234. 10.1007/s10964-012-9863-9867 23180070

[B13] Calmeiro L. Camacho I. Gaspar M. (2018). Life satisfaction in adolescents: the role of individual and social health assets. *Span. J. Psychol.* 21 1–8. 10.1017/sip.2018.24 30056810

[B14] Campbell A. (1981). *The Sense of Well-Being in America.* New York, NY: McGraw-Hill, 10.1093/sf/61.1.332

[B15] Casas F. (2011). Subjetive social indicators and child and adolescent well-being. *Child Indic. Res.* 4 555–575. 10.1007/s12187-010-9093-z

[B16] Casas F. Sarriera J. C. Abs D. Coenders G. Alfaro J. Saforcada E. (2011). Subjective indicators of personal well-being among adolescents. Performance and results for different scales in Latin-language speaking countries: a contribution to the international debate. *Child Indic. Res.* 5 1–28. 10.1007/s12187-011-9119-9111

[B17] Casas F. Sarriera J. C. Alfaro J. González M. Figuer C. Abs da Cruz D. (2014). Satisfacción escolar y bienestar subjetivo en la adolescencia: poniendo a prueba indicadores para su medición comparativa en Brasil, Chile y España. *Suma Psicol.* 21 70–80. 10.1016/S0121-4381(14)70009-8

[B18] Casas J. A. Ortega-Ruiz R. Del Rey R. (2015). Bullying: the impact of teacher management and trait emotional intelligence. *Br. J. Educ. Psychol.* 85 407–423. 10.1111/bjep.12082 26095169

[B19] Cenkseven-Önder F. Çolakkadioglu O. (2013). Decision-making and problem-solving as a well-being indicator among adolescents. *Educ. Res. Rev.* 8 720–727.

[B20] Choi D. Minote N. Sekiya T. Watanuki S. (2016). Relationships between trait empathy and psychological well-being in Japanese university students. *Psychology* 7 1240–1247. 10.4236/psych.2016.79126

[B21] Cohen J. Cohen P. West S. G. Aiken L. S. (2003). *Applied Multiple Regression/Correlation Analysis for the Behavioral Sciences*, 3rd Edn Mahwah, NJ: Lawrence Erlbaum Associates Publishers, 10.4324/9780203774441

[B22] Cummins R. A. Eckersley R. Lo S. K. Okerstrom E. Hunter B. Da-vern M. (2003). *Australian Unity Well-being Index: Cumulative Psychometric Record*. Report 9.0. Melbourne, VIC: Australian Centre on Quality of Life.

[B23] Curran T. Wexler L. (2017). School-based positive youth development: a systematic review of the literature. *J. Sch. Health* 87 71–80. 10.1111/josh.12467 27917486

[B24] Denham S. A. Wyatt T. M. Bassett H. H. Echeverría D. Knox S. S. (2009). Assessing social-emotional development in children from a longitudinal perspective. *J. Epidemiol. Commun. Health* 63 137–152. 10.1136/jech.2007.070797 19098138

[B25] Díaz-Aguado M. J. Martínez Arias R. Martín Babarro J. (2013). Bullying among adolescents in Spain. Prevalence, participant roles and characteristics attributable to victimization by victims and aggressors. *Rev. Educ.* 362 348–379. 10.4438/1988-592X-RE-2011-362-164

[B26] Elipe P. Ortega R. Hunter S. C. del Rey R. (2012). Perceived emotional intelligence and involvement in several kinds of bullying. *Psicol. Conduct.* 20 169–181.

[B27] Espelage D. L. Low S. K. Jimerson S. R. (2014). Understanding school climate, aggression, peer victimization, and bully perpetration: contemporary science, practice, and policy. *Sch. Psychol. Q.* 29 233–237. 10.1037/spq0000090 25198615

[B28] Evans C. B. R. Smokowski P. R. (2015). Theoretical explanations for bullying in school: how ecological processes propagate perpetration and Victimization. *Child Adolesc. Soc. Work J.* 33 365–375. 10.1007/s10560-015-0432-432

[B29] Extremera N. Rey L. (2018). Core self-evaluations are associated with judgments of satisfaction with life via positive but not negative affect. *Pers. Individ. Dif.* 130 112–116. 10.1016/j.paid.2018.03.054

[B30] Flaspohler P. D. Elfstrom J. L. Vanderzee K. L. Sink H. E. (2009). Stand by me: the effects of peer and teacher support in mitigating the impact of bullying on quality of life. *Psychol. Sch.* 46 636–649. 10.1002/pits.20404

[B31] Gademann A. M. Schonert-Reichl K. A. Zumbo B. D. (2010). Investigating validity evidence of the Satisfaction with Life Scale Adapted for children. *Soc. Indic. Res.* 96 229–247. 10.1007/s11205-009-9474-9471

[B32] Gelman A. Hill J. (2006). *Data Analysis Using Regression and Multilevel/Hierarchical Models.* Cambridge: Cambridge University Press, 10.1017/cbo9780511790942

[B33] Gilman R. Huebner S. (2003). A review of life satisfaction research with children and adolescents. *Sch. Psychol. Q.* 18 192–205. 10.1521/scpq.18.2.192.21858

[B34] Guerra N. G. Bradshaw C. P. (2008). Linking the prevention of problem behaviors and positive youth development: core competencies for positive youth development and risk prevention. *New Dir. Child Adolesc. Dev.* 122 1–17. 10.1002/cd.225 19021244

[B35] Harris M. A. Wetzel E. Robins R. W. Donnellan M. B. Trzesniewski K. H. (2018). The development of global and domain self-esteem from ages 10 to 16 for Mexican-origin youth. *Int. J. Behav. Dev.* 42 4–16. 10.1177/0165025416679744

[B36] Hinduja S. Patchin J. W. (2017). Cultivating youth resilience to prevent bullying and cyberbullying victimization. *Child Abuse Negl.* 73 51–62. 10.1016/j.chiabu.2017.09.010 28945996

[B37] Jackson V. Chou S. Browne K. (2017). Protective factors against child victimization in the school and community: an exploratory systematic review of longitudinal predictors and interacting variables. *Trauma Violence Abuse* 18 303–321. 10.1177/1524838015611675 26492892

[B38] Jolliffe D. Farrington D. P. (2006). Development and validation of the basic empathy scale. *J. Adolesc.* 29 589–611. 10.1016/j.adolescence.2005.08.010 16198409

[B39] Kaye-Tzadok A. Kim S. S. Main G. (2017). Children’s subjective well-being in relation to gender - What can we learn from dissatisfied children? *Child. Youth Serv. Rev.* 80 96–104. 10.1016/j.childyouth.2017.06.058

[B40] Konishi C. Miyazaki Y. Hymel S. Waterhouse T. (2017). Investigating associations between school climate and bullying in secondary schools: multilevel contextual effects modeling. *Sch. Psychol. Int.* 38 240–263. 10.1177/0143034316688730

[B41] Lachmann B. Sindermann C. Sariyska R. Y. Luo R. Melchers M. C. Becker B. (2018). The role of empathy and life satisfaction in internet and smartphone use disorder. *Front. Psychol.* 9:398. 10.3389/fpsyg.2018.00398 29636714 PMC5881138

[B42] Lent R. W. Taveira M. C. Sheu H. Singley D. (2009). Social cognitive predictors of academic adjustment and life satisfaction in Portuguese college students: a longitudinal análisis. *J. Vocat. Behav.* 74 190–198. 10.1016/j.jvb.2008.12.006

[B43] Lester L. Cross D. (2015). The relationship between school climate and mental and emotional wellbeing over the transition from primary to secondary school. *Psychol. Well Being* 5:9. 10.1186/s13612-015-0037-8 26516619 PMC4615665

[B44] Low S. Van Ryzin M. (2014). The moderating effects of school climate on bullying prevention efforts. *Sch. Psychol. Q.* 29 306–319. 10.1037/spq0000073 25089333

[B45] Lu C. Jiang Y. Zhao X. Fang P. (2019). Will helping others also benefit you? Chinese adolescents’ altruistic personality traits and life satisfaction. *J. Happiness Stud.* 1–19. 10.1007/s10902-019-00134-6

[B46] Lyons M. D. Huebner E. S. Hills K. J. (2016). Relations among personality characteristics, environmental events, coping behavior and adolescents’ life satisfaction. *J. Happiness Stud.* 17 1033–1050. 10.1007/s10902-015-9630-z

[B47] Martínez-Ferrer B. Moreno-Ruiz D. Amador L. V. Orford J. (2011). School victimization among adolescents. An analysis from an ecological perspective. *Psychosoc. Intervent.* 20 149–160. 10.5093/in2011v20n2a3

[B48] Méndez I. Ruiz-Esteban C. López-García J. J. (2017). Risk and protective factors associated to peer school victimization. *Front. Psychol.* 8:441. 10.3389/fpsyg.2017.00441 28382016 PMC5360713

[B49] Menesini E. Salmivalli C. (2017). Bullying in schools: the state of knowledge and effective interventions. *Psychol. Health Med.* 22 240–253. 10.1080/13548506.2017.1279740 28114811

[B50] Metzger A. Alvis L. M. Oosterhoff B. Babskie E. Syvertsen A. Wray-Lake L. (2018). The intersection of emotional and sociocognitive competencies with civic engagement in middle childhood and adolescence. *J. Youth Adolesc.* 47 1663–1683. 10.1007/s10964-018-0842-5 29572778

[B51] Miranda R. Oriol X. Amutio A. Ortúzar H. (2019). Bullying en la adolescencia y satisfacción con la vida: ¿puede el apoyo de los adultos de la familia y de la escuela mitigar este efecto? *Rev. Psicodidáct.* 24 39–45. 10.1016/j.psicod.2018.07.001

[B52] Moraleda M. González A. García-Gallo J. (2004). *Actitudes y Estrategias Cognitivas Sociales.* Madrid: TEA Ediciones.

[B53] Oliva A. Antolín L. Pertegal M. A. Ríos M. Parra A. Hernando A. (2011a). *Instrumentos Para la Evaluación del Desarrollo Positivo Adolescente y Los Activos Que lo Promueven.* Sevilla: Departamento de Psicología Evolutiva y de la Educación.

[B54] Oliva A. Reina M. Hernando A. Antolín L. Pertegal M. A. Parra A. (2011b). *Activos Para el Desarrollo Positivo y la Salud Mental en la Adolescencia.* Sevilla: Junta de Andalucía.

[B55] Olweus D. (1993). *Bullying at School: What We Know and What We Can Do.* Oxford: Blackwell, 10.1002/pits.10114

[B56] Olweus D. Breivik K. (2014). “Plight of victims of school bullying: The opposite of well-being,” in *Handbook of Child Well-being: Theories, Methods and Policies in Global Perspective*, eds Ben-Arieh A. Casas F. Frønes I. Korbin J. E. (Dordrecht: Springer), 2593–2616. 10.1007/978-90-481-9063-8_100

[B57] Ortega-Ruiz R. Del Rey R. Casa J. A. (2016). Evaluar el bullying y el cyberbullying validación española del EBIP-Q y del ECIP-Q. *Psicol. Educ.* 22 71–79. 10.1016/j.pse.2016.01.004

[B58] Overbeek G. Zeevalkink H. Vermulst A. Scholte R. H. (2010). Peer victimization, self-esteem, and ego resilience types in adolescents: a prospective analysis of person-context interactions. *Soc. Dev.* 19 270–284. 10.1111/j.1467-9507.2008.00535.x

[B59] Palacios E. G. Echaniz I. E. Fernández A. R. De Barrón I. C. O. (2015). Personal self-concept and satisfaction with life in adolescence, youth and adulthood. *Psicothema* 27 52–58. 10.7334/psicothema2014.105 25633770

[B60] Pertegal M. A. Hernando A. (2015). “El contexto escolar como promotor del desarrollo positivo adolescente,” in *Desarrollo Positivo Adolescente*, ed. Oliva A. (Madrid: Síntesis), 59–80.

[B61] Pertegal M. A. Oliva A. Hernando A. (2010). Los programas escolares como promotores del desarrollo positivo adolescente. *Cult. Educ.* 22 53–66. 10.1174/113564010790935169 26187923

[B62] Rey L. Extremera N. Sánchez-Álvarez N. (2019). Clarifying the links between perceived emotional intelligence and well-being in older people: pathways through perceived social support from family and friends. *Appl. Res. Qual. Life* 14 221–235. 10.1007/s11482-017-9588-9586

[B63] Rezapour M. Khanjani N. Mirzai M. (2019). Exploring associations between school environment and bullying in Iran: multilevel contextual effects modeling. *Child. Youth Serv. Rev.* 99 54–63. 10.1016/j.childyouth.2019.01.036

[B64] Rosenberg M. (1965). *Society and the Adolescent Self-image.* Princeton, NJ: Princeton University Press, 10.1126/science.148.3671.804

[B65] Salguero J. M. Fernandez-Berrocal P. Balluerka N. Aritzeta A. (2010). Measuring perceived emotional intelligence in the adolescent population: psychometric properties of the trait meta-mood scale. *Soc. Behav. Pers. Int. J.* 38 1197–1209. 10.2224/sbp.2010.38.9.1197

[B66] Salovey P. Mayer J. D. Goldman S. L. Turvey C. Palfai T. P. (1995). “Emotional attention, clarity, and repair: Exploring emotional intelligence using the Trait Meta-Mood Scale,” in *Emotion, Disclosure, & Health*, ed. Pennebaker J. W. (Washington, DC: American Psychological Association), 125–154. 10.1037/10182-006

[B67] Sánchez-Álvarez N. Extremera N. Fernández-Berrocal P. (2015). Maintaining life satisfaction in adolescence: affective mediators of the influence of perceived emotional intelligence on overall life satisfaction judgments in a two-year longitudinal study. *Front. Psychol.* 6:1892. 10.3389/fpsyg.2015.01892 26834654 PMC4714630

[B68] Saphire-Bernstein S. Taylor S. E. (2013). “Close relationships and happiness,” in *Oxford Handbook of Happiness*, eds Boniwell I. David S. A. Ayers A. C. (Oxford: Oxford University Press), 821–833. 10.1093/oxfordhb/9780199557257.013.0060

[B69] Sapouna M. Wolke K. D. (2013). Resilience to bullying victimization: the role of individual, family and peer characteristics. *Child Abuse Negl.* 37 997–1006. 10.1016/j.chiabu.2013.05.009 23809169

[B70] Savahl S. Montserrat C. Casas F. Adams S. Tiliouine H. Benninger E. (2018). Children’s experiences of bullying victimization and the influence on their subjective well-being: a multinational comparison. *Child Dev.* 90 414–431. 10.1111/cdev.13135 30207591

[B71] Scales P. Benson P. Oesterle S. Hill K. Hawkins D. Pashak T. (2016). The dimensions of successful Young adult development: a conceptual and measurement framework. *Appl. Dev. Sci.* 20 150–174. 10.1080/10888691.2015.1082429 30344455 PMC6176765

[B72] Schipper M. Petermann F. (2013). Relating empathy and emotion regulation: do deficits in empathy trigger emotion dysregulation? *Soc. Neurosci.* 8 101–107. 10.1080/17470919.2012.761650 23327297

[B73] Shanafelt T. D. West C. Zhao X. Novotny P. Kolars J. Habermann T. (2005). Relationship between increased personal well-being and enhanced empathy among internal medicine residents. *J. Gen. Intern. Med.* 20 559–564. 10.1007/s11606-005-0102-108 16050855 PMC1490167

[B74] Siegel R. S. La Greca A. M. Harrison H. M. (2009). Peer victimization and social anxiety in adolescents: prospective and reciprocal relationships. *J. Youth Adolesc.* 38 1096–1109. 10.1080/17470919.2012.761650 19636774

[B75] Suldo S. M. Thalji-Raitano A. Hasemeyer M. Gelley C. D. Hoy B. (2013). Understanding middle school students life satisfaction: does school climate matter? *Appl. Res. Qual. Life* 8 169–182. 10.1007/s11482-012-9185-9187

[B76] Sung Y. H. Chen L. M. Yen C. F. Valcke M. (2018). Double trouble: the developmental process of school bully-victims. *Child. Youth Serv. Rev.* 91 279–288. 10.1016/j.childyouth.2018.06.025

[B77] Taylor R. Oberle E. Durlak J. Weissberg R. (2017). Promoting positive youth development through school-based social and emotional learning interventions: a meta-analysis of follow-up effects. *Child Dev.* 88 1156–1171. 10.1111/cdev.12864 28685826

[B78] Tolan P. Ross K. Arkin N. Godine N. Clark E. (2016). Toward an integrated approach to positive development: implications for intervention. *Appl. Dev. Sci.* 20 214–236. 10.1080/10888691.2016.1146080 23144705

[B79] Ttofi M. M. Farrington D. P. Lösel F. Loeber R. (2011). Do the victims of school bullying tend to become depressed later in life? A systematic review and meta-analysis of longitudinal studies. *J. Aggress. Confl. Peace Res.* 3 63–73. 10.1108/17596591111132873

[B80] Valois R. F. Zulling K. J. Huebner E. S. Drane J. W. (2009). Youth developmental assets and perceived life satisfaction: is there a relationship? *Appl. Res. Qual. Life* 4 315–331. 10.1007/s11482-009-9083-9089

[B81] Varela J. J. Sirlopú D. Melipillán R. Espelage D. Green J. Guzmán J. (2019). Exploring the influence school climate on the relationship between school violence and adolescent subjective well-being. *Child Indic. Res.* 1–16. 10.1007/s12187-019-09631-9

[B82] Voight A. Nation M. (2016). Practices for improving Secondary School Climate: a systematic review of the research literature. *Am. J. Commun. Psychol.* 58 174–191. 10.1002/ajcp.12074 27535489

[B83] Waasdorp T. E. Bradshaw C. P. Leaf P. J. (2012). The impact of schoolwide positive behavioral interventions and supports on bullying and peer rejection. A Randomized Controlled Effectiveness Trial. *Arch. Pediatr. Adolesc. Med.* 166 149–156. 10.1001/archpediatrics.2011.755 22312173

[B84] Yoon J. Sulkowski M. L. Bauman S. A. (2016). Teachers’ responses to bullying incidents: effects of teacher characteristics and contexts. *J. Sch. Violence* 15 91–113. 10.1080/15388220.2014.963592

[B85] Zych I. Beltrán-Catalán M. Ortega-Ruiz R. Llorent V. J. (2018). Social and emotional competencies in adolescents involved in different bullying and cyberbullying roles. *Rev. Psicodidáctica* 23 86–93. 10.1016/j.psicod.2017.12.001 30006668

[B86] Zych I. Farrington D. P. Ttofi M. M. (2019). Protective factors against bullying and cyberbullying: a systematic review of meta-analyses. *Aggress. Violent Behav.* 45 4–19. 10.1016/j.avb.2018.06.008

